# Effect of Cover Plate on the Ballistic Performance of Ceramic Armor

**DOI:** 10.3390/ma14010001

**Published:** 2020-12-22

**Authors:** Miao Sun, Wuxiong Cao, Diqi Hu, Nana Zhang, Runqiang Chi

**Affiliations:** 1 School of Astronautics, Harbin Institute of Technology, Harbin 150080, China; sunmiaols@hit.edu.cn (M.S.); wuxiong.cao@hit.edu.cn (W.C.); hudiqi1991@hit.edu.cn (D.H.); 2 School of Civil Engineering, Southeast University, Nanjing 210096, China; 230208581@seu.edu.cn

**Keywords:** cover plate, ceramic armor, interface defeat, ballistic performance, transition impact velocity

## Abstract

The interface defeat and dwell can effectively improve the ballistic performance of ceramic armors under high velocity impact of long rod projectiles. Confinement conditions along both axial and radial directions of ceramic armors can affect these behaviors. With the aim of giving an insight into the effect of cover plate thickness and connection mode of cover plates with confining tubes on these behaviors, numerical simulations were performed in which the confined silicon carbide (SiC) targets with cover plates were impacted by tungsten rods. The pressure on the surfaces of SiC targets with fixed cover plates are compared to that with free cover plates, showing that the plates fixed with the confining tubes can produce higher pressure by way of wedging. With the increase in cover plate thickness, the dwell duration of the tungsten rods on the ceramic interface gradually grows. In addition, the upper and lower limits of transition impact velocities for the SiC targets with cover plates in different connection modes (i.e., free or fixed) were obtained and analyzed. The results show that the increase rate of the transition velocity region for the cover plate with the fixed-mode is relatively stable and lower than with the free-mode. On this basis, the fixed cover plate contributes higher ballistic performances to the SiC target than the free cover plate. It is also noteworthy that the size of transition velocity region does not enlarge linearly with the increase in cover plate thickness due to the slow growth of the upper limit. Accordingly, thickness thresholds exist, which are 5 mm and 6 mm for the fixed and free cover plates, respectively. Considering the ballistic performance and economy, the cover plate with the thickness ranging from 3 mm to 5 mm, i.e., 1.5~2.5 times of the tungsten rod diameter, is ideal for the structural dimensions in this paper.

## 1. Introduction

Ceramics have a wide range of potential applications in the domain of military defense because of their high hardness, high strength, and low density. Under high-velocity impact, a typical phenomenon (i.e., interface dwell) on a ceramic surface can be generally observed, which means that the projectile is eroded on the surface of the ceramic and flows out radically without penetration [1]. Meanwhile, interface defeat happens when the projectile is eroded completely. Generally, transition between the interface defeat and penetration can be marked by the transition velocities, and penetration and interface defeat occur above and below the transition velocities, respectively. Within the transition velocity region, the dwell–penetration transition, which means dwell followed by penetration, occurs. Therefore, transition velocities and dwell duration are the key values to evaluate the ballistic performance of ceramics [2].

Typically, two approaches are exploited to enhance the ballistic performance of ceramics, i.e., exerting pre-stress or adding confinement on the ceramic. Massive experiments and numerical simulations [3,4,5,6] suggest that the application of pre-stress can greatly improve the ballistic performance of ceramics. However, the pre-stress conditions are usually difficult for practical applications. Comparatively, adding confinement is more achievable [7], including a lateral confinement and a cover plate. Savio et al. [8] used the standard depth of penetration (DOP) method to evaluate the ballistic performance of the B_4_C-based ceramic target against 7.62 mm armor-piercing projectiles. It was found that the penetration depth with the reinforced steel lateral confinement was reduced by 34% compared to the unreinforced sample. Comparing with the experimental results provided by Doyoyo [9], it was noted that when the material impedance of the lateral confinement was close to that of the ceramic, the ceramic exhibits relatively high ballistic performance. However, the effect of lateral confinement was related to the ceramic target size and the impact velocity. When the ceramic was large enough or the impact velocity was very fast, the lateral confinement effect could be ignored.

In addition, a cover plate placed in front of the ceramic can be used as a buffer. Hauver et al. [10] conducted a series of DOP tests on Al_2_O_3_. They founded that ceramic with a cover plate had better ballistic performance. Ning et al. [11] confirmed that after, adding a cover plate, the starting position of the ceramic target damage changed from the front surface to the back surface [12]. Therefore, the cover plate could reduce the peak value of impact loading on the ceramic target and the damage caused by the direct impact of the projectile. Sarva et al. [13] observed that with a cover plate, the projectiles were eroded more severely and had much greater mushrooming. It was believed that the ceramic powder constrained by the cover plate was the most essential reason for this phenomenon. These results indicated that the cover plate changed the damage process of the ceramic, thereby enhancing its ballistic performance. It was observed in experiments that when interface dwell occurred, the projectile pieces could form a radial flow, rushing into the gap between ceramic and the cover plate. The potential effect of radial flow should not be ignored, especially under different confinement conditions of the cover plate, i.e., free or fixed. Surprisingly, Lundberg’s study [14] found that when the cover plate was fixed to the lateral confining tube, the effect of radial flow might be enhanced. However, only a small amount of the literature [15,16] has discussed the role of radial flow up to now. Zhai et al. [17] showed that with a fixed cover plate, the DOP was reduced by 10% compared to that with a free plate, but a further explanation for this phenomenon was not provided.

Although some studies demonstrate that the transition impact velocity of SiC with a cover plate could be increased from 800 m/s to 1500 m/s [18], cover plates with small thickness have no significant effect on interface dwell and penetration [19]. Consequently, it will greatly increase the mass of ceramic armor while blindly increasing the thickness of the cover plate. However, a cover plate with enough thickness is still necessary to prevent it from bulging, indicating that an optimal thickness value exists for the cover plate. Many new cover materials have been developed, such as fiber-reinforced polymer (FRP) [20], glass fiber composite materials [21,22] and boron carbide composite materials [23], while their experimental results need to be further discussed. In general, metal cover plates are more commonly used at present; Zhai et al. [17] confirmed that the performance of the armored steel cover plate is the best, followed by copper. Luo et al. [24] examined the effect of metal cover plates on ballistic performance of silicon carbide subjected to large-scale tungsten projectiles, showing that the harder steel cover was beneficial for the ballistic performance. Therefore, it is vitally important to explore the effect of a cover plate with different connection modes (i.e., free or fixed) and varying thickness on the interface defeat and dwell.

In this paper, numerical simulations are carried out to analyze the effect of fixed and free cover plates with different thicknesses on the ballistic performance of a silicon carbide (SiC) target using Finite Element Method (FEM)/Smoothed Particle Hydrodynamics (SPH) models in AUTODYN^®^. The damage process of the SiC target at different impact velocities, the change of surface pressure near to the impact point of the SiC target, the upper and lower limit of transition velocities, and dwell duration are discussed. These studies can provide some reference for the structural design and mechanism analysis of ceramic armor.

## 2. Numerical Simulations

**Figure 1:**
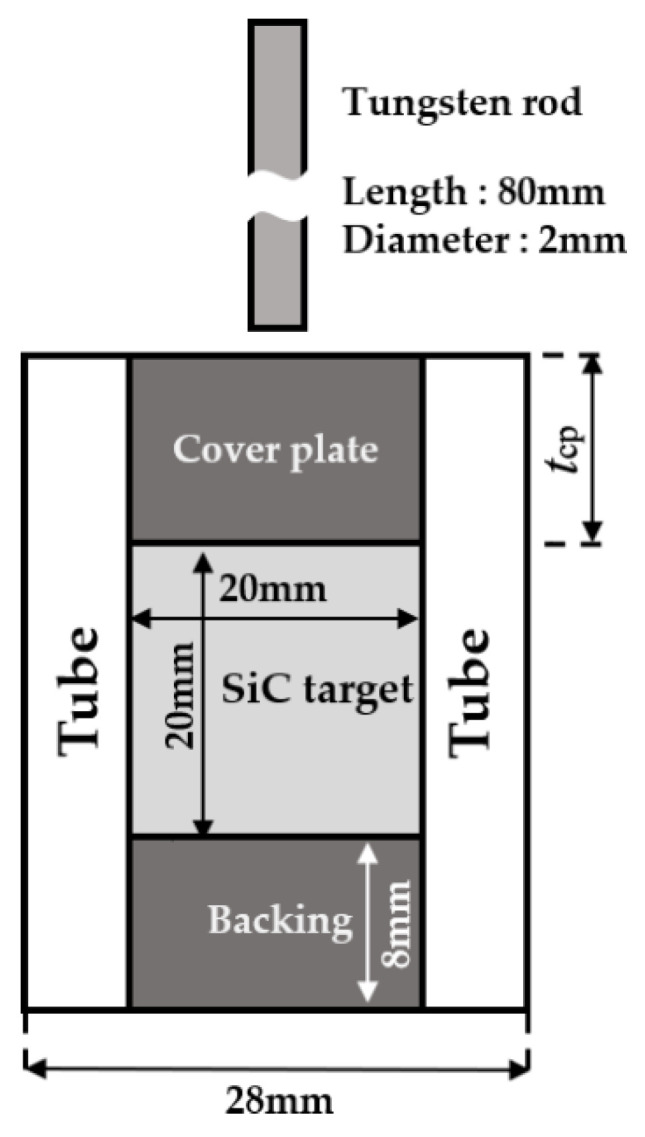
Structure geometry of the confined SiC target used in the experiments. In the present numerical simulations, thickness of the cover plate (*t*_cp_) ranges from 3 mm to 8 mm at an interval of 1 mm.

The confined SiC target refers to that used in the experiments carried out by Lundberg et al. [6]. The structural geometries consist of an SiC target, a maraging steel (Mar 350) tube, tempered steel (SIS 2541-3, comparable to AISI/SAE 4340) cover and backing plates. The target structure is impacted by a cylindrical tungsten rod (2 mm in diameter and 80 mm in length) at different velocities. The thickness of the cover plate (denoted by *t*_cp_), ranges from 3 mm to 8 mm at an interval of 1 mm. The sketch of an SiC target, tube, cover plate, backing and tungsten rod is shown in Figure 1.

### 2.1. Numerical Model

**Figure 2:**
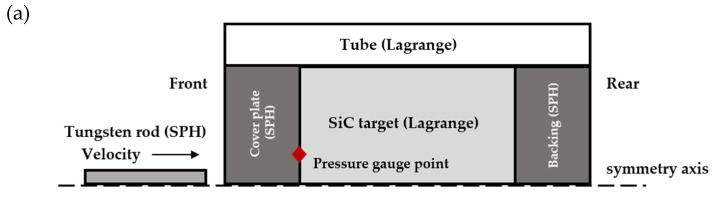

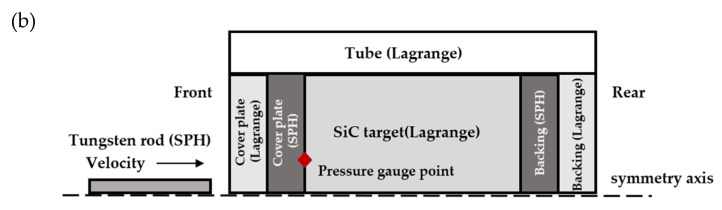
2D axisymmetric model for the rod and the target, showing part of the numerical model. The pressure gauge point is set on the SiC target surface. (**a**) Free cover plate. (**b**) Fixed cover plate.

Two-dimensional (2D) axisymmetric models of the SiC target and tungsten rod were developed using AUTODYN^®^, as shown in Figure 2. Axisymmetric Lagrangian elements with four nodes and one integration point were used to discretize SiC targets and steel tubes. The rod is discretized by the smooth particle hydrodynamics (SPH) method. Regarding the cover plate not fixed to the tube, both the cover and backing plates are modeled by the SPH method. For the cover plate fixed to the tube, the cover and backing plates are composed of two parts, i.e., the outer and inner parts, which are modeled using the Lagrangian method and SPH method, respectively, as shown in Figure 2. These two parts are joined together using the “JOIN module” on their interface. In all cases, the backing plates are joined to the tubes. The SPH particle size in the tungsten rod, the cover and backing plates is 0.125 mm, which is the same as that used by Quan et al. [25]. The Lagrangian element sizes in the SiC target, and the outer part of the cover and backing plates are 0.15 mm and 0.125 mm, respectively. A pressure gauge point is set on the surface of the SiC target with a distance of 2 mm away from the symmetry axis.

The reason for considering a Lagrangian–SPH model for the cover plates joined to tubes is as follows: an erosion algorithm is used to remove the elements experiencing large distortions. If the inner part of a cover plate is modeled using the Lagrangian method, a zero-pressure void will be introduced when an element close to the SiC target is eroded, allowing SiC material at the interface to expand towards the void and lose pressure as it expands. As the strength and damage of the SiC material are dependent on the pressure, then the pressure drop can lead to lower strength and premature failure of the SiC. In the SPH domain, there is no grid restriction, and the particles are permitted to move freely under deformation. The inner part of cover plate in contact with SiC modeled using the SPH method can maintain the existing pressure and then strength in the front of the SiC target.

### 2.2. Material Models and Constants

**Figure 3:**
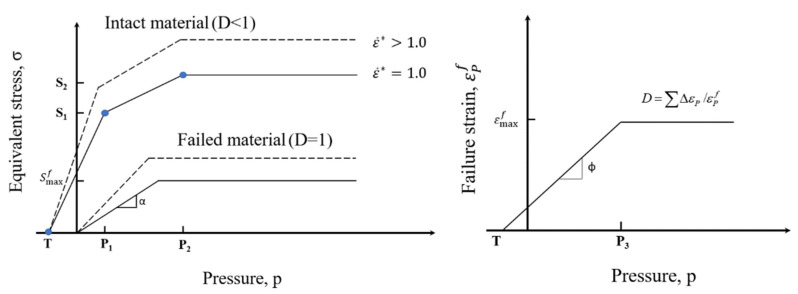
Description of the Johnson–Holmquist-1(JH-1) constitutive model for ceramics.

The Johnson–Holmquist-1 (JH-1) model is pervasively used in simulations for investigation of the long rod projectile impacting on the ceramic interface of B_4_C and SiC ceramics, the numerical results are consistent with the experimental results [17,25,26,27,28]. According to our previous work [28], JH-1 model represents SiC behavior well, and it is used in this paper. The original form of the JH-1 model presented by Johnson and Holmquist [26] is summarized in Figure 3, the JH-1 material model constants used for SiC are listed in Table 1 [28]. It shows that the available strength *σ* is dependent on the pressure *P*, the dimensionless strain rate ${\dot{\varepsilon }}^{*}=\dot{\varepsilon }/{\dot{\varepsilon }}_{0}$ and the damage *D* (0≤D≤1). The maximum tensile hydrostatic pressure which the material can experience is *T*. *S*_1_ and *S*_2_ are the strengths of the intact material at compressive pressures *P*_1_ and *P*_2_, respectively. When the material fails, the slope of the strength of the failed material is given by α. The maximum failure strength is $S_{\max}^{f}$, when ${\dot{\varepsilon }}^{*}=1.0$. The strain rate constant is *C*. σ_0_ is the available strength at ${\dot{\varepsilon }}^{*}=1.0$, then the strength at other strain rates is expressed as

(1) $$\sigma =\sigma_{0}(1.0+C\ln{\dot{\varepsilon }}^{*}),$$

The accumulated damage for failure is written as

(2) $$D=\sum \Delta \varepsilon_{P}/\varepsilon_{P}^{f},$$

where $\Delta \varepsilon_{P}$ is the increment in equivalent plastic strain during a cycle of integration, $\varepsilon_{P}^{f}$ is the equivalent plastic strain at failure under a constant pressure *P*. Referring to the right portion of Figure 3, the material cannot undergo any plastic strain at the maximum hydrostatic tension *T*, but it increases to $\varepsilon_{p}^{f}=\varepsilon_{\max}^{f}$ at a compressive pressure of *P = P*_3_. *P*_3_ and $\varepsilon_{\max}^{f}$ are the damage constants. Then the failure strain is simply defined as

(3) $$\varepsilon_{p}^{f}=\frac{\varepsilon_{\max}^{f}}{\left(P_{3}+T\right)}(P+T),$$

The hydrostatic pressure before failure is depicted as

(4) $$P=K_{1}\mu +K_{2}\mu^{2}+K_{3}\mu^{3},$$

where *K*_1_, *K*_2_ and *K*_3_ are constants, and $\mu =V_{0}/V-1$ for current volume *V*, initial *V*_0_. For tensile pressures, the above equation is replaced by $P=K_{1}\mu$. Bulking can occur after failure. This provides an additional incremental pressure ΔP

(5) $$P=K_{1}\mu +K_{2}\mu^{2}+K_{3}\mu^{3}+\Delta P,$$

The additional incremental pressure ΔP is calculated as

(6) $$\Delta P=-K_{1}\mu_{f}+\sqrt{{\left(K_{1}\mu_{f}\right)}^{2}+2\beta K_{1}\Delta U},$$

where $\mu_{f}$ is μ at failure, and β is the ratio of the elastic energy loss converted to potential hydrostatic energy. ΔU is the loss elastic internal energy.

The Johnson–Cook (JC) constitutive and failure models are used for the tungsten rod [29] and steel 4340 cover and backing plates [30]. The material properties and parameters are listed in Table 2 and Table 3. The Von Mises constitutive model and effective plastic strain failure model are used for the steel Mar 350 tube [25]. The Shear modulus, yield stress, and failure strain are 77 GPa, 2.6 GPa, and 0.4, respectively. The equation of state (EOS) of shock is used for the tungsten alloy, and the linear EOS is used for steel 4340 and steel Mar 350. The constants of EOS are listed in Table 4.

### 2.3. Validation of the Numerical Model

**Figure 4:**
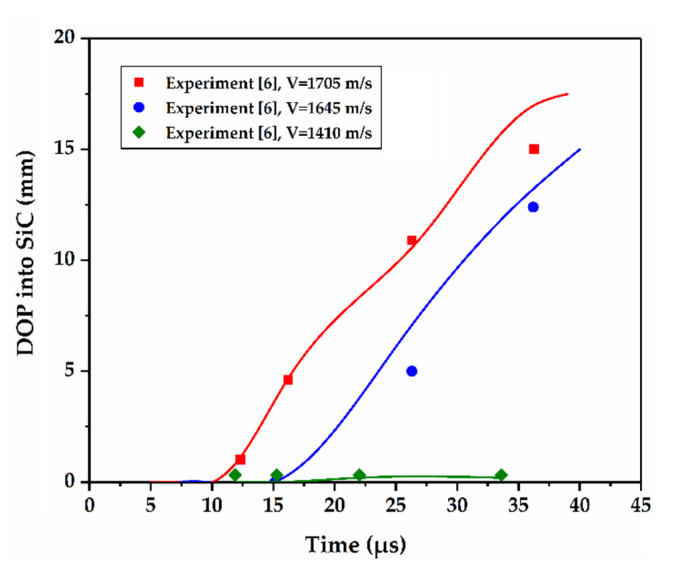
Comparison of depth of penetration (DOP) results obtained from simulations with experimental measurements [6].

Three experiments presented by Lundberg et al. [6] were simulated, the impact velocities of which are 1410 m/s, 1645 m/s and 1705 m/s, respectively. In the simulations, the interface defeat, dwell–penetration transition, and penetration are observed. Variation of the DOP in SiC targets with time is compared with experimental measurements. As shown in Figure 4, the errors are all within an acceptable range, and are also close to the simulation results of our previous work [28].

## 3. Results and Discussion

In order to analyze the effect of the cover plate on the ballistic performance, numerical simulation results of tungsten rods impacting the confined SiC targets were performed. Two connection modes between the cover plate and tube, i.e., free- and fixed-modes, were modeled in the numerical model. In addition, numerical results of interface defeat, dwell–penetration transition and penetration for different thickness of cover plates were obtained. The damage process of SiC target, dwell duration, and transition velocities will be discussed as follows.

### 3.1. Damage Mechanism of SiC Target with Different Connection Modes

**Figure 5:**
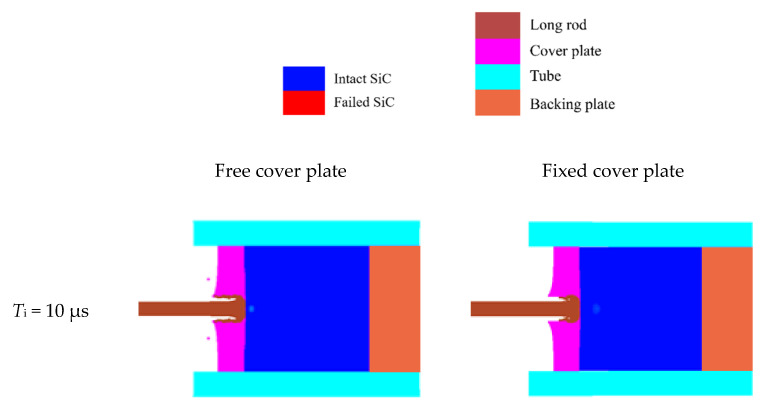

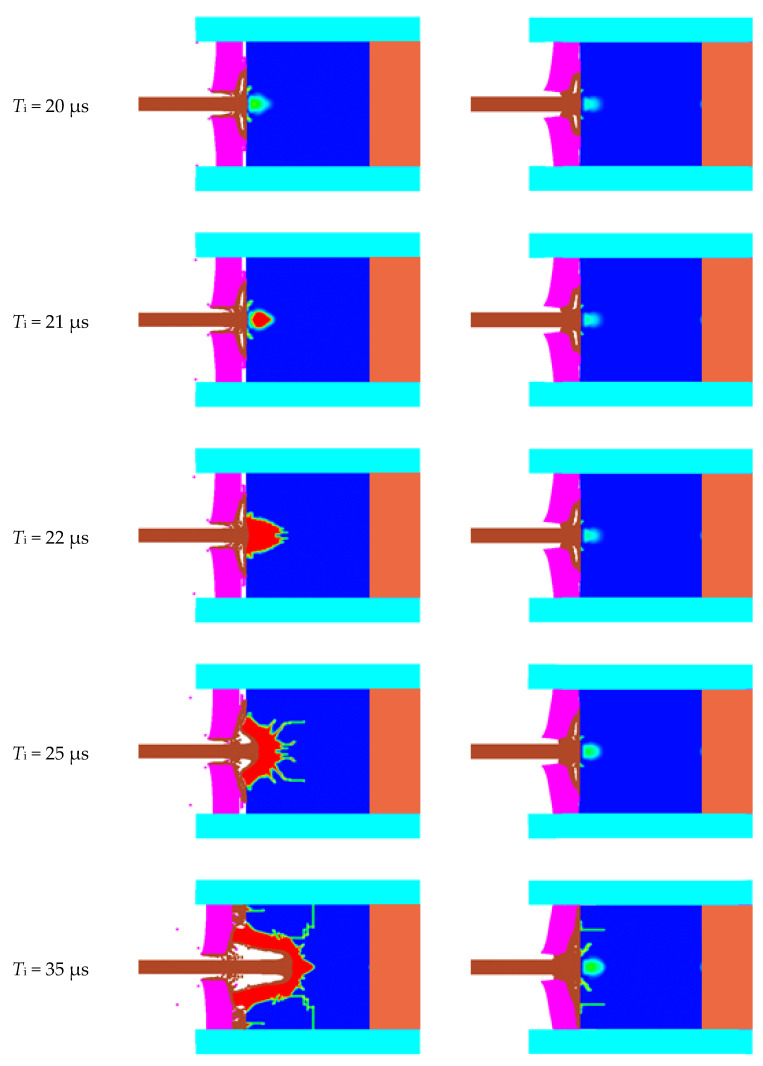
Damage of confined SiC target with the free and fixed cover plates at different time the. The cover plate thickness is 4 mm and the impact velocity is 1250 m/s.

By way of illustrations, the SiC target with both free and fixed cover plates (4 mm in thickness) impacted by the tungsten rod at the velocity of 1250 m/s are analyzed. The impact process at different times (denoted by *T*_i_, which is defined as the time after the tungsten rod starts to move from its initial position) from 10 μs to 35 μs are shown in Figure 5. When *T*_i_ = 10 μs, the tungsten rod completely penetrates the cover plate and makes contact with the SiC target, and the initial damage position in the SiC target with the fixed cover plate is deeper than that with free cover plate.

It is obvious that the interface dwell of the tungsten rod is generated for both the free- and fixed- modes for the time in the range of 10 μs to 21 μs. Simultaneously, radial flow of the tungsten rod material is produced, which flows into the gap between the cover plate and the SiC surface. It is worth noting that the cover plate is squeezed gradually away from the SiC target for the free-mode, whereas it only bends and deforms for the fixed-mode. At 21 μs, the subsurface of the SiC target with the free cover plate fails, and the damage extends from the internal structure to the surface when *T*_i_ = 22 μs, indicating that the dwell–penetration transition occurs. Regarding the SiC target with the fixed cover plate, no complete damage occurs during the observed period, and the state of interface dwell is always maintained.

**Figure 6:**
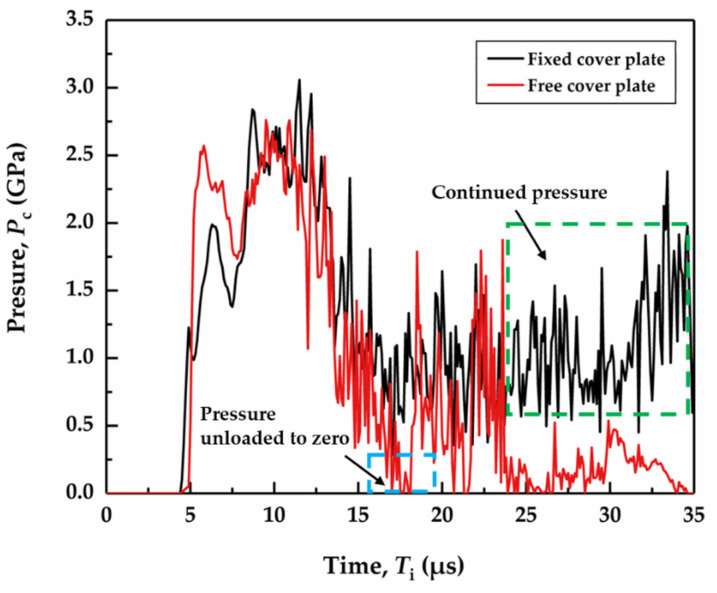
Time history of pressure at the gauge point on the SiC target surface with the fixed and free cover plate. The cover plate thickness is 4 mm and the impact velocity is 1250 m/s.

Compared to free-mode, the enhancement of the ballistic performance of the SiC target for the fixed-mode can be attributed to the radial flow caused after the interface dwell. Since the radial flow is constrained by the cover plate, it will keep pressure (denoted by *P*_c_) on the SiC surface. Time histories of pressure at the gauge point 2 mm away from the impact point on the SiC target surface with both fixed and free cover plates are shown in Figure 6. The tungsten rod begins to make contact with the cover plate at 4 μs, the initial pressure peak on the surface of SiC target with the fixed cover plate is 2.00 GPa, lower than that for the free cover plate, i.e., 2.55 GPa. At the impact time of 10 μs, the tungsten rod is in contact with the SiC target, and the surface stress reaches the maximum value. The peak pressure on the SiC target surface with fixed cover plate is slightly higher than that for the free-mode, showing 3.75 GPa and 3.00 GPa, respectively. For the impact time in the range of 10 μs to 20 μs when interface dwell occurs, the cover plate is deformed or even lifted as a result of radial flow, and *P*_c_ begins to decrease.

Regarding the free-mode, *P*_c_ is completely unloaded for the first time at about 17 μs. As the strength of the SiC target is positively correlated with the hydrostatic pressure, therefore its strength decreases rapidly, and the internal damage area expands quicky. For the fixed-mode, although *P*_c_ slightly decreases, it always oscillates around 1 GPa. The greater the hydrostatic pressure is, the higher the strength of the SiC target is. This continuous pressure helps the SiC target to maintain its strength, leading to the less change of the internal damage area. In addition, the pressure *P*_c_ is completely unloaded the second time at 21 μs for the free-mode cover plate, and the interface dwell is transformed into penetration. At 30 μs, the pressure peak of the SiC target surface with fixed cover plate shows an upward trend, because of the increasing amount of radial flow in the gap between the cover plate and the SiC surface, which in turn applies higher force on the SiC target surface.

However, the hydrostatic pressure of the SiC target will be reduced when the radial flow interacts strongly with the tube, which was confirmed by Lundberg et al. [31]. The use of a large-diameter target will increase the time for the radial flow to reach the tube, leading to the improvement of the ballistic performance.

### 3.2. Dwell Duration

**Figure 7:**
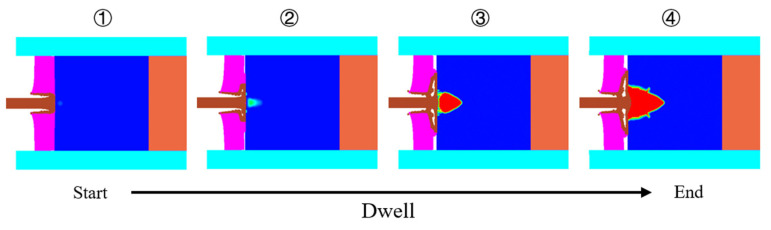
Illustration of the dwell process at the impact velocity of 1200 m/s (4 mm thick cover plate with the free-mode).

Targeting the investigation of the effect of the cover plate thickness as well as the connection mode on the dwell time, numerical simulations were conducted, in which the cover plate thickness (t_cp_) was set as 3 mm, 4 mm, 5 mm, 6 mm, 7 mm, and 8 mm, respectively, and the impact velocity was 1200 m/s, 1400 m/s and 1600 m/s. Figure 7 illustrates a complete dwell process at the impact velocity of 1200 m/s. It starts when the tungsten rod contacts the SiC target surface (see the first image) and ends when the internal damage extends to the SiC target surface (see the fourth image). The dwell time (*T*_d_) is defined as the time after dwell starts, and the *T*_du_ means the duration of dwell.

**Figure 8:**
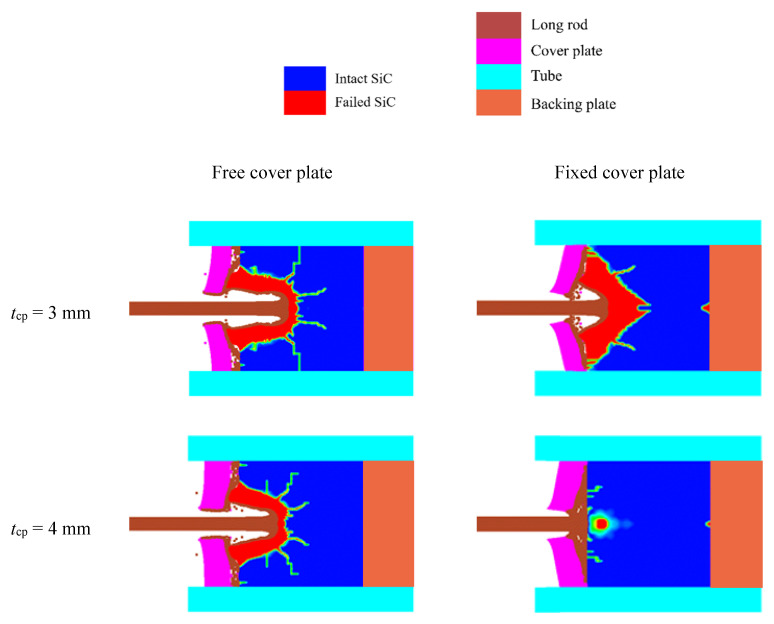

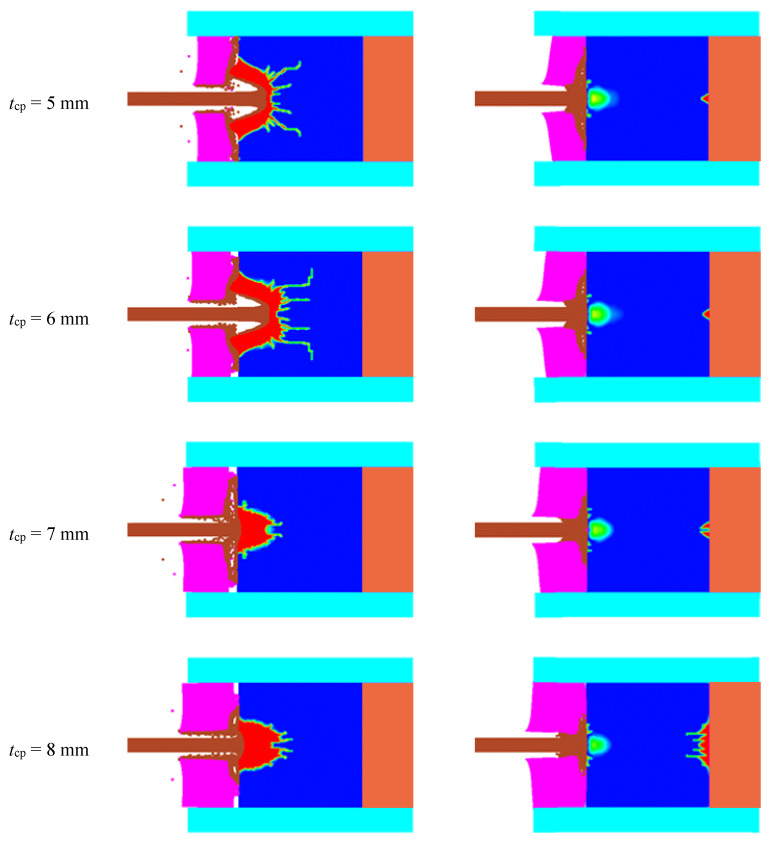
Computed results for tungsten rods impacting confined SiC targets with different cover plates. The impact velocity is 1400 m/s and the dwell time, *T*_d_, is 26 μs.

Representatively, damage of SiC target at the dwell time of 26 μs under the impact velocity of 1400 m/s for the cover plates with different connection modes and varying thicknesses are demonstrated in Figure 8. The tube, plugs and rod are shown in different colors, respectively; it is obviously that there is less penetration in SiC targets for the fixed-mode than that for the free-mode. At the dwell time of about 26 μs, the dwell–penetration transition begins to occur on the SiC target surfaces when the free cover plate is 7 mm and 8 mm in thickness. Tungsten rods penetrate into the SiC targets when the cover plate is 3 mm~6 mm thick. As for the fixed-mode, penetration is only observed when the thickness of cover plate is 3 mm, and the tungsten rods are in the dwell process on cover plates thicker than 3 mm. Generally, the penetration decreases as cover plate thickness increases from 3 mm to 8 mm. All these phenomena are due to the amount of dwell that occurs.

Table 5 and Table 6 summarize the dwell duration in all simulation cases. Under the high-velocity impact at 1200 m/s, projectiles are completely defeated when the fixed and free cover plates have thicknesses of 3 mm~8 mm and 6 mm~8 mm, respectively. The dwell duration gradually grows with the increasing thickness of the free cover plate in the range of 3 mm~5 mm. It is noteworthy that the connection between the cover plate and the tube can greatly enhance the interface dwell performance of the SiC target and reduce the cover plate thickness by about 50%.

**Figure 9:**
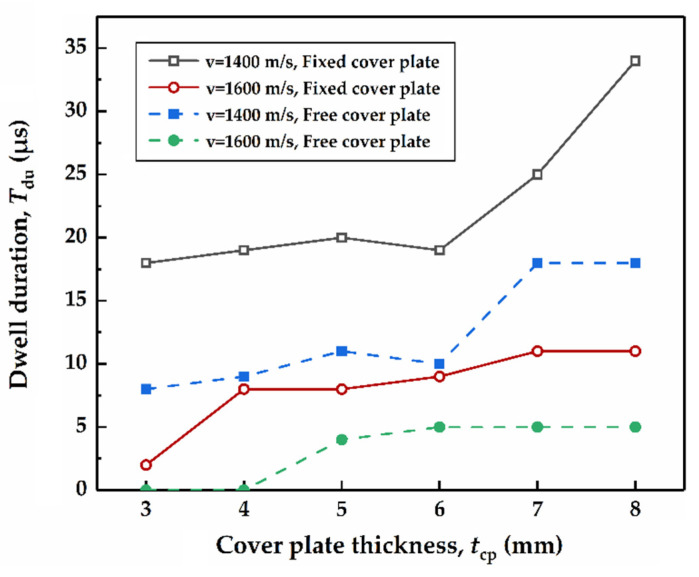
Simulated dwell duration in cases in which the impact velocities are 1400 m/s and 1600 m/s.

The variation of dwell duration for the cover plates with different connection modes and varying thicknesses are demonstrated in Figure 9. The impact velocities are 1400 m/s and 1600 m/s, respectively, it can be founded that the dwell duration decreases as the impact velocity increases. For the cover plates with the thicknesses of 3 mm and 4 mm, when the impact velocity is 1600 m/s, interface defeat is generated for the fixed-mode, while dwell-penetration transition occurs for the free-mode. As for the cover plates with other thickness, the average dwell duration for the fixed-mode is about two times of that for the free-mode. Obviously, connecting the cover plate to the tube can greatly increase the dwell duration. For both free-and fixed-modes, when the impact velocity is 1400 m/s, dwell duration increases slowly as the cover plate thickness changes from 3 mm to 6 mm, then it grows rapidly as the thickness varies from 6 mm to 8 mm. Similarly, this phenomenon was also observed in experiments when the ratio of the cover plate thickness to the rod diameter was 0.25~1 and the impact velocity was medium [32].

**Figure 10:**
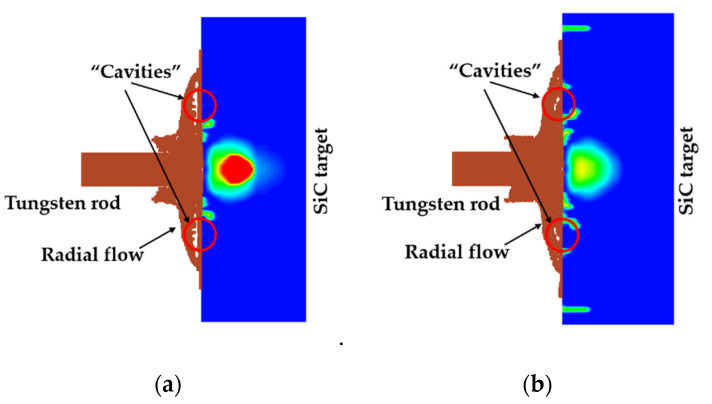
Comparison of the radial flow pattern in cases with the 6 mm and 7 mm fixed cover plates at the same dwell time of 18 μs. The impact velocity is 1400 m/s. The cover plate and the tube are not shown. (**a**) *t*_cp_ = 6 mm. (**b**) *t*_cp_ = 7 mm.

Figure 10 shows the radial flow pattern of the rod fragments for the cover plate thicknesses of 6 mm and 7 mm at the same dwell time, respectively. Cavities are observed in the radial flow, which are larger when *T*_cp_ = 6 mm. They disappear more quickly when the cover plate is 7 mm thick. It seems that the thicker cover plate can prevent the cavity from happening. Combined with the analysis of the radial flow effect in the preceding Section 3.1, the disappearance of “cavities” seems to be beneficial to the pressure maintenance of the ceramic surface.

In addition, as the impact velocity increases to a higher value, 1600 m/s, the dwell duration increases slowly with increasing cover plate thickness. In other words, when the impact velocity is relatively high and close to the upper limit of transition impact velocity, the effect of both the cover plate thickness and connection mode are not significant even if the initial pressure in the SiC target can be reduced. The specific reasons will be described in the following Section 3.3.

### 3.3. Transition Impact Velocity

**Figure 11:**
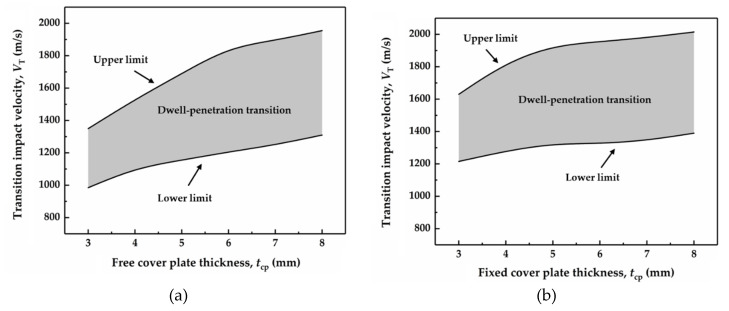
Transition impact velocity versus cover plate thickness. The grey areas correspond to the interval between the lower and upper limits of transition impact velocity. (**a**) Free cover plate and (**b**) fixed cover plate.

Three typical phenomena, i.e., penetration, interface dwell, and interface defect, are observed from the damage cloud image of the SiC target, when the impact velocity of tungsten rod is in the range of 900 m/s~2200 m/s with an interval of 5 m/s. Accordingly, the region (i.e., the grey area) of transition impact velocity between the interface defeat and penetration is determined, as shown in Figure 11. Penetration occurs when the damage extends from the internal to the surface of SiC target. The lower limit of transition impact velocity corresponds to the maximum velocity at which the tungsten rod is completely defeated by the ceramic target, but no penetration emerges. In addition, when the dwell duration of the tungsten rod is approximately 1 μs under a specific impact velocity in these simulations, and the damage immediately extends from the internal to the surface of SiC target after this dwell, then this velocity is defined as the upper limit of transition impact velocity.

As demonstrated in Figure 11, the upper and lower limits of transition impact velocity can be obtained, respectively, when the cover plates with different thicknesses and connection modes are impacted with high velocity. For the cover plate with the free-mode, the increase rates of both the upper and lower limits are rapid and then slow down with the increase in cover plate thickness, as displayed in Figure 11a. The size of the transition velocity region gradually increases and then remains stable. Regarding the cover plate with the fixed-mode, the upper limit of transition velocity increases rapidly when the thickness of the cover plate gradually changes from 3 mm to 5 mm, then the increase rate slows down, as shown in Figure 11b. The increase rate of the lower limit is relatively stable, and the fastest growth rate occurs when the thickness changes from 3 mm to 4 mm. In addition, the transition velocity region (i.e., the grey area) gradually enlarges with the increase in cover plate thickness in the range 3 mm~7 mm, while this region narrows when the thickness continues to grow from 7 mm to 8 mm. It is noteworthy that the fixed cover plates’ even relatively thin will cling to the surface of the ceramics and not is easily lifted by radial flow (seen in Figure 8), thus their upper and lower limits are more stable when compared to that with the free-mode.

Compared to the cover plate with the free-mode, both the upper and lower limits for the cover plate with the fixed-mode are higher regardless of the thickness, while the size of the transition velocity region is only larger when the cover plate is 3 mm, 4 mm, and 5 mm in thickness. In addition, it can be seen that the growth rate of the transition velocity region is the largest for the fixed cover plate with the thickness of 4 mm (equal to that of the tube). Then, the increase rate of the transition velocity region for the cover plate with the free-mode is higher than that with the fixed-mode, which decreases slightly. Therefore, the size of the transition velocity region does not enlarge linearly with the increase in cover plate thickness due to the slow growth of the upper limit. Accordingly, thickness thresholds exist, which are 5 mm and 6 mm for the fixed and free cover plates, respectively.

**Figure 12:**
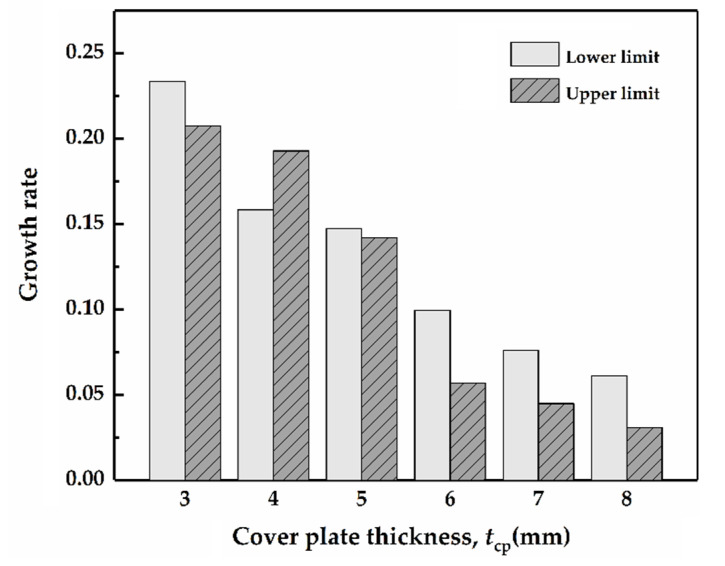
The growth rate of the upper and lower limits of transition impact velocity when the cover plate connection mode changes from free to fixed.

The increase rates of the upper and lower limits for the cover plate with both the free- and fixed- modes are compared, as shown in Figure 12, which stand out when the thicknesses are 3 mm, 4 mm and 5 mm, respectively. As for the cover plate with the fixed-mode (3 mm in thickness), the growth rates for the lower and the upper limits are 20.1% and 23.3%, respectively, when compared to that with the free-mode. For the plate with the thickness of 4 mm, the increase rate of the upper limit reaches about 19.3%, exceeding that of the lower limit (15.8%). Regarding the 5 mm plate, the increase rate of the lower limit is close to the upper limit, and these are 14.7% and 14.2%, respectively. When the thickness exceeds 5 mm, the increase rates of the upper and lower limits decrease gradually in a similar way. Accordingly, the differences between the fixed- and free-mode cover plates can guide the structural design. Areal density is an important index to evaluate the performance of protective structure, the increase in the unnecessary mass of the protective structure will lead to a decrease in the protection benefit. Therefore, the cover plate with the thickness ranging from 3 mm to 5 mm, i.e., 1.5~2.5 times of the projectile diameter, is ideal for the current structural dimensions. With the increase in impact velocity, the transition from interface defeat to penetration occurs; the pressure holding effect of radial flow decreases gradually, because the defeat interface directly determines the formation of the radial flow of the projectile. The earlier the penetration occurs, the lower the amount of radial flow is, which in turn reduces the pressure holding effect and further promotes the occurrence of penetration. Although the fixed-mode can enhance the pressure holding effect of radial flow, it plays a small role under relatively high impact velocity.

## 4. Conclusions

In this paper, numerical results of tungsten rods impacting SiC targets are presented to study the effects of cover plates on the ballistic performance of confined SiC targets. The damage mechanism of SiC targets with different cover plate connection modes, dwell duration, and transition impact velocity are discussed. The results of this study are summarized as follows:

(1) The fixed cover plates change the damage process of the SiC target compared to the free cover plate. The radial flow generated during dwell introduces a higher pressure in the SiC target through wedging into the gap between the fixed cover plate and the SiC target surface, thereby delaying the SiC damage evolution. The ballistic performance of the SiC target represented by dwell duration and the upper and lower limits of transition impact velocity is better for the fixed-mode than that for the free-mode.
(2) The dwell duration and the upper and lower limits of transition impact velocity increase with increasing cover plate thickness generally. Considering ballistic performance and economy, the cover plate with the thickness ranging from 3 mm to 5 mm, i.e., 1.5~2.5 times the tungsten rod diameter, is ideal for the structural dimensions in this paper.
(3) The effects of cover plate thickness and its connection mode become more insignificant when the impact velocity exceeds a certain value.

## Figures and Tables

**Table 1:** JH-1 material model constants for SiC [28].

Parameters	SiC
Density, *ρ*_0_ (kg/m^3^)	3215
Bulk modulus, *K*_1_ (GPa)	220
Pressure constant, *K*_2_ (GPa)	361
Pressure constant, *K*_3_ (GPa)	0
Bulking factor, *β*	1.0
Shear modulus, *G* (GPa)	193
Hugoniot elastic limit, HEL (GPa)	11.7
Intact Strength Constant, *S*_1_ (GPa)	7.1
Intact Strength Constant, *P*_1_ (GPa)	2.5
Intact Strength Constant, *S*_2_ (GPa)	12.2
Intact Strength Constant, *P*_2_ (GPa)	10.0
Strain rate constant, *C*	0.009
Maximum fracture strength, $S_{\max}^{f}$ (GPa)	1.3
Failed strength constant, *α*	0.4
Hydrostatic tensile limit, T* (GPa)	−0.75
Principal tensile failure stress, *T*_f_ (GPa)	1.3
Damage constant, $\varepsilon_{\max}^{f}$	0.5
Damage Constant, *P*_3_ (GPa)	99.75
Fracture energy, *G*_f_ (J/m^2^)	37.3

**Table 2:** Johnson–Cook (JC) strength model constants for the tungsten alloy [29] and steel 4340 [30].

Parameters	Tungsten Alloy	Steel 4340
Shear modulus, *G* (GPa)	160	77
Static yield stress, *A*’ (GPa)	1.506	0.75
Strain hardening constant, *B*’(GPa)	0.177	0.51
Strain hardening exponent, *n*	0.12	0.26
Strain rate constant, *C*’	0.016	0.014
Reference strain rate, ${\dot{\varepsilon }}_{0}$	1	1
Thermal softening exponent, *m*	1	1.03
Melting temperature, *t*_m_ (K)	1723	1793

**Table 3:** JC failure model constants for the tungsten alloy and steel 4340 [30].

Parameters	Tungsten Alloy	Steel 4340
Damage Constant, *D*_1_	0	0.05
Damage Constant, *D*_2_	0.33	3.44
Damage Constant, *D*_3_	−1.5	−2.12
Damage Constant, *D*_4_	0	0.003
Damage Constant, *D*_5_	0	0.61

**Table 4:** Equation of state (EOS) constants for the tungsten alloy [29], steel 4340 [30] and steel Mar 350 [25].

Parameters.	Tungsten Alloy	Steel 4340	Steel Mar 350
EOS	Shock	Linear	Linear
Density, *ρ*_0_ (kg/m^3^)	17,600	7830	8080
Bulk Modulus, *K*_1_ (GPa)	285	159	140
Gruneisen constant	1.54	−	−
Parameter *C*_1_ (m/s)	4029	−	−
Parameter *S*	1.237	−	−
Reference temperature, *t*_0_ (K)	300	300	293
Specific heat, *C*_t_ (H/kg K)	134	477	−

**Table 5:** Dwell duration in cases with the fixed cover plate.

Velocity (m/s)	*T*_du_ (μs)					
	*t*_cp_ = 3 mm	*t*_cp_ = 4 mm	*t*_cp_ = 5 mm	*t*_cp_ = 6 mm	*t*_cp_ = 7 mm	*t*_cp_ = 8 mm
1200	−	−	−	−	−	−
1400	18	19	20	19	25	34
1600	2	8	8	9	11	11

“−” means complete interface defeat.

**Table 6:** Dwell duration in cases with the free cover plate.

Velocity (m/s)	*T*_du_ (μs)					
	*t*_cp_ = 3 mm	*t*_cp_ = 4 mm	*t*_cp_ = 5 mm	*t*_cp_ = 6 mm	*t*_cp_ = 7 mm	*t*_cp_ = 8 mm
1200	14	15	21	−	−	−
1400	8	9	11	10	18	18
1600	×	×	4	5	5	5

“−” means complete interface defeat, and “×” means penetration without dwell.

## Data Availability

The experimental measurements presented in Figure 4 are openly available in [web of science] at [10.1016/S0734-743X(99)00152-9], Ref. number [6]. The parameters of material model presented in Table 2, Table 3 and Table 4 are openly available in [web of science] at [10.1016/j.ijimpeng.2006.09.011, 10.1016/0013-7944(85)90052-9], reference number [25,30]. The data in Ref. [29] can be found in [google scholar] with the link [apps.dtic.mil].
